# Social connections and participation among people with mild cognitive impairment: barriers and recommendations

**DOI:** 10.3389/fpsyt.2023.1188887

**Published:** 2023-07-05

**Authors:** Di Zhu, Abdullah Al Mahmud, Wei Liu

**Affiliations:** ^1^Swinburne University of Technology, Hawthorn, VIC, Australia; ^2^Beijing Normal University, Beijing, China

**Keywords:** social participation, people with MCI, dementia, social connections, technology

## Abstract

**Objective:**

Social connections and participation are essential for individuals with mild cognitive impairment (MCI) to prevent the progression of cognitive decline and enhance their overall well-being. However, existing research has primarily focused on environmental barriers, overlooking personal factors and the interconnected nature of these barriers. Moreover, there is a lack of understanding regarding social connections and participation challenges specific to people with MCI in low- and middle-income countries. Therefore, this study aimed to explore the barriers that hinder social connections and participation among people with MCI in China and investigate opportunities to design appropriate supportive interventions.

**Methods:**

Thirty-one people with MCI (13 males and 18 females; mean age = 82.74 years, SD = 7.69; mean MoCA score = 21.26, SD = 2.44) and 13 caregivers were recruited to attend focus groups. In addition, 10 therapists were interviewed using a semi-structured interview guide. Focus groups and interviews were audio-recorded, transcribed, and analyzed using a framework analysis approach.

**Results:**

We found that the ability to build social connections among people with MCI is affected by mobility challenges, intensive grandparenting responsibilities, availability of suitable activities, and feelings of exclusion in a closed-minded community environment. Personal reasons, such as lower social efficacy, inability to find suitable social activities, and intensive family responsibilities, discourage people with MCI from social participation. The digital literacy of people with MCI depends on their motivation to learn and use digital tools; people with MCI who live in a community have a higher digital literacy than those living in care centers. The motivation to achieve digital literacy is affected by perceived benefits, costs of technology use, and social influence.

**Conclusion:**

Multidimensional initiatives are needed to address barriers to social connections, participation, and technology adoption among individuals with MCI. This includes organizing and engaging in social activities, promoting awareness and education on the importance of social participation, and exploring technology-based interventions to improve memory and storytelling abilities. These efforts can create a supportive environment and empower individuals with MCI to participate actively in social interactions, enhancing their overall well-being.

## 1. Introduction

Mild cognitive impairment (MCI) is a condition characterized by objectively identifiable cognitive impairment that is not severe enough to support a diagnosis of early dementia or significant functional impairment but results in subtle deficiencies observed by the individual or their relatives (1, 2). MCI and dementia are closely related, with an annual transition rate from mild impairment to dementia of 10–15 and 50% within 5 years (3). People with MCI face numerous challenges in daily life due to cognitive deficits and emotional changes, which lead to a reduced ability to function socially (4). Previous studies have reported that older adults with better health are more engaged in social activities (5), and cognitive status may impact mobility and mood (6). Mobility is closely related to the social exclusion of older adults (7). Therefore, compared to healthy older adults, individuals with MCI face a higher risk of social exclusion and further cognitive decline.

Social connection encompasses a person’s closest relationships, good friends, and communities, and is often referred to as a “circle of connections” (8). Social participation for people with MCI refers to their involvement in social activities that require interpersonal interactions outside the home (9). Despite the cognitive changes experienced by individuals with MCI (10), engaging in high-level social participation has been shown to prevent or slow cognitive decline (11, 12), maintain significant social roles (13), improve quality of life (14), reduce negative influences on others (15), and decrease social isolation (16). Low social participation and social isolation among people with MCI can lead to further cognitive decline (17, 18) and exacerbate the impact on cognitive function (19).

Social participation behavior may be influenced by cognitive impairment, as individuals with MCI may experience changes in their daily routines that their family and friends may not readily perceive. Tasks that were once easily accomplished may become challenging, resulting in a loss of interest in activities. For example, individuals with MCI may stop reading, visiting friends (due to fear of becoming disoriented and unable to understand conversations), engaging in hobbies, and participating in professional activities. However, they typically have specific activities in which they wish to continue their mastery. Effective interventions that help people with MCI improve their social participation are crucial. Furthermore, higher levels of social participation have been associated with lower odds of developing MCI, which is not primarily attributed to loneliness in low- and middle-income countries, including China (20). Therefore, several interventions have been developed to support the social connections and participation of people with MCI. Interventions aimed at supporting older adults in social connections and participation can be categorized into group (21), and one-on-one interventions (22). One-on-one interventions may include occupational therapy (23) and home visits (24). However, one-on-one interventions have limited efficiency because they involve one therapist supporting each individual at a time. Therefore, group interventions have been developed to address efficiency challenges. Dickens reviewed interventions to support older adults and proposed two types of group interventions: those providing activities and support (25). Activities aim to create new possibilities for social connections and social participation or improve specific abilities, such as using technology (26) and cognitive abilities (27). Support interventions include group therapy (28), counseling, and discussion (29). Most group interventions involve therapists who support or organize the interventions to ensure effective delivery.

However, a shortage of qualified therapists for people with MCI in low- and middle-income countries, such as China, has been identified (30). Consequently, many researchers have turned to technology-based interventions to support social connections and participation for people with MCI. Social connections, including social network quality, size, frequency, and closeness, are the main elements of social participation (31). Various programs have been designed, including cognitive rehabilitation programs (32, 33), educational programs (34), telecommunication systems (35), social robots (36, 37), and self-management systems (38), to improve the social participation of people with MCI or dementia. Numerous therapies, such as animal-assisted interventions (39), sensory interventions (40, 41), educational programs (42), and cognitive-behavioral interventions (43), have been explored to enhance social participation. Several studies have highlighted the potential of technology-based interventions to support social participation for people with MCI (38, 44) due to their feasibility, accessibility, cost-effectiveness, and utility (45–47). Technology allows interventions to expand beyond the hospital setting to the home environment and reduces the workload of therapists (48).

While technology-based interventions offer opportunities to improve social participation, they also present challenges. Digital inclusion directly impacts the quality of life of Chinese older adults, with a quantitative study in China showing that quality of life is a linear function of disability status, attitude toward technology, and digital inclusion. The study also emphasized the importance of older adults’ attitudes toward technology in promoting digital inclusion (49). Technology facilitates older adults’ lives by providing remote channels for contact with others and acting as an external memory aid to overcome memory issues. For example, older adults reported positive experiences with virtual home health care visits, which met their complex needs (50). Technology-based social memory aids have also been shown to reduce social stress for older adults with memory concerns (51). Additionally, technology commonly supports people with MCI in social participation and instrumental activities (52). Although older adults, including those with cognitive impairment, may encounter usability issues, they have reported positive experiences and feasibility (53, 54). Overcoming these usability issues requires motivation from older adults, with or without cognitive impairment (55). However, as cognitive function declines, older adults may face increased difficulty in using technology (56). Therefore, it can be argued that technology could help older adults, especially those with MCI, improve their social participation.

However, current studies often consider social participation as a secondary goal and neglect the holistic perspective of improving social participation. For example, researchers may aim to improve cognitive abilities through cognitive rehabilitation and find psychosocial effects (33). However, they may limit their research scope to improving social participation directly linked to cognitive abilities. To develop technology-based interventions that support social participation, opportunities such as influencing factors should be further explored.

Numerous barriers may prevent people with MCI from participating in social activities, which are crucial aspects of successful aging across cultures (57). For instance, a study revealed that mood problems are a risk factor for dementia progression, and the researchers proposed that enlarging social networks may slow down this progression (58). Barriers faced by older adults with vision and hearing impairments are mainly environmental, such as accessibility and societal attitudes. Individual barriers include the type of impairment and attitudes toward impairment (59). While it is valuable to investigate factors that may affect the progression from MCI to dementia, further exploration is needed to understand how these factors influence social participation. The barriers to social participation for people with MCI are often more invisible and broader in nature. However, individuals with mild cognitive impairment differ from lonely older adults or those with vision and hearing impairments. Social participation is influenced by cultural background, the type of activities, and the type of relationships. For example, compared to older adults in the USA, older adults in China tend to avoid engaging in political activities (60), and fewer older Chinese adults participate in religious activities compared to older adults in Western countries (61). The phenomenon of intergenerational family support is widespread in Asian countries such as Indonesia (62) and China (63). Grandparenting is a complex social role that can have dual effects (64). While some researchers consider grandparenting as a new social role that creates opportunities for social participation (65), others argue that the heavy burden of grandparenting during older age may harm cognitive abilities (66). Although attempts have been made to address the barriers to social participation, the factors that may hinder social participation among those with MCI in China remain unknown. We could tailor a more effective intervention design for people with MCI after identifying the challenges, opportunities, and internal factors by obtaining feedback from this population. Therefore, we conducted seven focus groups to explore the experiences of people with MCI and their caregivers and ten one-on-one interviews with therapists to identify professional perspectives on the influences related to social participation among people with MCI. This study aimed to answer the following research questions.

1. What barriers hinder social connections, social participation, and the use of technology among people with MCI?
2. What are the recommendations for overcoming these barriers?
3. What interconnections exist between social connections, social participation, and the use of technology for people with MCI?

## 2. Methodology

### 2.1. Methods

In this study, we employ closeness, contact frequency, and social circle size to define the quality of social connections (31). Since people with MCI may lack explanations for their social participation experiences, a focus group setting was selected because it can provide opportunities for the participants to supplement each other’s ideas of others, especially in terms of motivations and reasons (67). Focus groups were used to engage and consult with people with MCI and their caregivers. People with MCI, and caregivers were gathered due to the limited time availability of therapists. Accordingly, we adopted semi-structured interviews to engage with this group (67). This study focused on identifying factors that may impede or motivate people with MCI regarding their social activities and provides recommendations for overcoming the identified barriers.

### 2.2. Participants

This research aims to identify the barriers to social connections and social participation among individuals with MCI, considering both those living in the community and those residing in care centers. Although these two groups may have different modes of social participation, they both encounter barriers due to cognitive decline. This study sought to determine intervention opportunities that can be effectively applied to both groups by investigating the common barriers to social connections and participation. To achieve this, two groups of individuals with MCI were recruited: those living in the community and those residing in care centers. Purposive sampling was employed to recruit people with MCI and their caregivers. The researcher contacted the managers of the care center, and a social working service company in Tiantongyuan community. These two partners are experts in psychology who facilitated participant recruitment. Moreover, a social working service company manager helped recruit people with MCI and conducted the Montreal Cognitive Assessment (MoCA) (68). We asked this manager to provide an information sheet to people with MCI and their caregivers, thus inviting people to participate. Purposive and snowballing sampling was used to recruit therapists. We proactively visited therapists and asked them to invite other qualified therapists to participate in this research. Moreover, we posted flyers on the wall of hospitals and care centers.

The inclusion criteria for people with MCI were those clinically diagnosed with MCI living in Beijing’s urban area aged 65 years or over without visual or hearing disabilities and with sufficient reading abilities (primarily digital interfaces). In addition, the participants were required to have a caregiver (family member or friend) willing to accompany them to the focus group. Participants were excluded if they had any significant neurological condition, such as a stroke or brain injury, since this would likely affect daily functioning and risk being a confounding factor in our analysis.

Regarding caregivers, the inclusion criteria were working as an informal caregiver for 1 year or longer or as a formal caregiver for 3 years or longer. Informal caregivers were generally relatives, including spouses and children. Formal caregivers were mainly nurses. Caregiver activities involved providing supervision, support, and assistance with daily activities and accompanying people with MCI as necessary.

The inclusion criteria for therapists were a master’s degree or above and experience working with people with MCI for 3 years or more. They were either occupational or clinical therapists.

This study was conducted according to the guidelines established by the National Statement on Ethical Conduct in Human Research (2018) and approved by the Swinburne University Human Research Ethics Committee (Reference: 20226525–10,539). We recruited 31 people with MCI (see Table 1; 13 male, 18 female; mean age = 82.74, range = 69–95 years; mean MoCA score = 21.26, range 18–26; SD = 2.44), 13 caregivers, and 10 therapists. The researcher DZ distributed the consent forms to the participants from the relevant partner institutions. Participants returned the signed consent form by sending an email or letter or handing the consent forms to DZ or a contactor in the partner institution in person.

**Table 1:** Demographic characteristics of research participants.

Characteristic	Group	*n*	Percent %
Gender	Male	13	41.93%
	Female	18	58.06%
Age (Average = 82.74, SD = 7.69)	65–69	1	3.23%
	70–74	5	16.13%
	75–79	5	16.13%
	80–84	7	22.58%
	85–90	7	22.58%
	Above 90	6	19.35%
Educational background	Bachelor’s degree	9	29.03%
	Junior college	2	6.45%
	Secondary specialized school	7	22.58%
	High school	3	9.68%
	Junior high school	5	16.13%
	Primary school	5	16.13%

### 2.3. Materials and settings

We conducted six online focus groups, one face-to-face focus group (in a discussion room in the community), and online one-on-one interviews (see Table 2). The focus group involving people with MCI and their caregivers lasted 90 min (four people with MCI and four caregivers per group). Focus groups for people with MCI and their caregivers covered four thematic issues (see Appendix): (1) People with MCI’s daily routines; (2) social connections; (3) social participation; and (4) community-based activities. Semi-structured interviews with therapists lasted 60 min each and focused on strategies and suggestions for using technology-based intervention programs. We asked therapists about the following behaviors, barriers, and recommendations for people with MCI (see Appendix): (1) instrumental activity challenges; (2) social connections; (3) social participation; and (4) community-based activities.

**Table 2:** Focus group participants’ information.

Variable	Number of focus groups							Total
	1	2	3	4	5	6	7	
Number of people with MCI	4	4	6	4	4	5	4	31
Mean age (years)	84.0	87.3	75.0	89.5	84.8	82.4	80.3	82.7
Number of caregiver	2	2	2	2	2	2	1	13
Gender %female	50.0	75.0	83.3	25.0	75.0	40.0	50.0	58.1

### 2.4. Data collection and analysis

The data analysis followed a thematic analytical approach (69) and took steps necessary for framework analysis (70). The COREQ guidelines for reporting the study outcomes were followed throughout (71). We used an audio recorder to record the focus groups and interviews. We then compared the automatic audio transcription and checked this verbatim against the audio recording to comprehensively review all transcripts, correcting any mistakes. For example, researcher DZ interviewed Therapist 1, and researcher RH would subsequently listen to the recording and review the transcripts. This approach allowed us to become familiar with the interview results of other researchers. After finalizing the Chinese transcripts, we sent them to all caregivers for review and copy approval. We then used DeepL^1^ to translate the Chinese text into English. The English transcript was reviewed by other researchers highly qualified in English reading and translation. As a rule, each researcher participated or reviewed transcripts at least once.

After acquiring robust familiarization with the transcripts, we selected two offering the most information and analytical potential: one focus group discussion and one one-to-one interview. Two researchers individually proposed the thematic code for every argument, attitude, behavior, barrier, and facilitator of social participation. Then, based on each coding outcome, we discussed each section collaboratively to define the final and more nuanced code headings and critically examined the code description. The code and its description formed the provisional basis of our analytical framework. DZ analyzed all transcripts, and RH analyzed half of the transcripts; the average consistency generated in the NVivo software was 0.825 (kappa), with overage agreement at 99.77%. We applied this framework during the data analysis process. If the researcher found that the transcript deviated from the codes, all three researchers discussed the situation and either identified a new code or clustered the unique instances with existing codes. We charted the transcript into the framework matrix based on the analytical framework outlined in Table 3. We categorized codes into themes and subthemes. We used ‘FG1-P2’ to represent focus group and patient numbers.

**Table 3:** Key themes and subthemes.

**Theme**	**Subthemes/codes**	**Participants group n/ratio**			**Example quote**
		**People with MCI *N* = 31**	**Caregiver *N* = 13**	**Therapist *N* = 10**	
Social connections	Lack of socialization and suitable activities	4/12.9%	3/23.3%	5/50%	*I do not want to participate in some activities. I think those activities are not suitable for me [FG3-P1]*
	Mobility challenges and required support	0/0	5/38.5%	5/50%	*The older adults with mobility problems, we will push the wheelchair for a walk. If their children come, they will be taken to a further place [FG1-C2]*
	Intensive grandparenting responsibilities	1/3.2%	0/0	1/10%	*My son has a 10-year-old 2nd and 3rd grader at home doing homework every day, so if you spend more time on him, you will not have time to do these activities. [FG3-P4]*
	Feeling excluded in a close-minded community environment	1/3.2%	1/7.7%	3/30%	*Some old adults are sometimes not sober (unclear thinking and strange behavior), he also wants to come to participate, but the sober mind of the elderly, he dislikes too much, there may be some of those aspects of the reason [FG6-P3]*
Social participation	Lack of motivation to overcome the challenges of social participation	4/12.9%	8/61.5%	4/40%	*There is no special need. I can stay indoors for a month [FG3-P4]*
	Lower social efficacy and higher performance expectation	5/16.1%	2/15.4%	8/80%	*I wanted to join the dance team before, but I was afraid that my zero-base performance would drag the team down [FG1-P2]*
	Impaired storytelling ability	3/9.7%	3/23.3%	3/30%	*Sometimes, I cannot retell clearly to my husband what I experienced today. The more worried I am, the more I cannot explain what just happened [FG5-P2]*
	Insufficient ability to perform independently in singing and dancing	11/35.5%	3/23.3%	9/90%	*It depends on the people with MCI where he is troubled; if it’s the kind of memory decline, his short-term memory this thing is very troubled, then we go to help him to do the relevant together with the attention to tell him some memory methods, to teach him to use some compensatory measures to learn to use that kind of simplified memory, and then some memory methods [Therapist 7]*
Technology for social connections and participation	Lack of motivation for independent use of mobile phones	5/6.4%	1/7.7%	5/50%	*I need help with that. I learned this when there was a group of college students visited us; they taught us how to use mobile phone [FG1-P2]*
	Negative social influence of using technology	4/12.9%	2/15.4%	5/50%	*But now the things on this phone are also quite complicated; some people have ulterior motives to carry out unreasonable propaganda, I am often criticized by my daughter for believing this [FG1-P3]*

## 3. Findings

### 3.1. Social connections

Family members are the primary connections of people with MCI, especially those who live in care centers. Most participants (*n* = 22, 71%) reported having no friends to contact since they had moved away or their friends had passed away. Some participants (*n* = 5, 16.1%) reported having over ten friends, and they all lived in the community. The two groups share the same activities but in a different location: a community or care center. Phone calls were the main people with MCI maintained social connections (*n* = 24, 77.4%). Some used the WeChat calling function (*n* = 11, 35.5%). People with MCI called family members approximately twice a week, whereas they called friends twice a month on average. Moreover, the duration of telephone communications was short: most participants (*n* = 20, 65%) reported a call duration of approximately 5 min. Fewer participants (*n* = 3, 9%) reported a duration longer than 1 h. Therefore, many people with MCI have (or at least exhibit) limited opportunities to communicate with others. People with MCI with opportunities to meet friends in person reported making arrangements with friends to visit the park (*n* = 7, 22.5%) or a favorite restaurant (*n* = 5, 16.1%) or meeting up with people when traveling (*n* = 5, 16.1%).

#### 3.1.1. Lack of socialization and suitable activities

People with MCI may evaluate the activities to determine whether they match their interests, available time, physical conditions, expected benefits, and expected performance. Expected benefits include improving physical health, not easy to catch a cold, a sense of fulfillment, and recognition of personal ability. Some people with MCI (*n* = 4, 12.9%) have many criteria regarding the type of activities they are willing to engage in and the people they are interested in meeting. As suggested by half of the therapists (*n* = 5, 50%), the decision to participate in activities depends on the personality of the individual with MCI, which is difficult to change. Thus, people with MCI may isolate themselves, only attend social activities that meet strict criteria, and show unwillingness to try new things.

*‘I do not want to participate in some activities. I think those activities are not suitable for me; for example, I will not go to the square dance since my body does not coordinate.’ [FG3-P1].*

Some people with MCI are afraid that their performance may affect others in the group or that a bad performance may trigger ridicule.

*‘I wanted to join the dance team before, but I was afraid that my zero-base performance would drag the team down.’ [FG1-P2].*

One potential reason cause of reluctance is personality resulting from MCI or introverted characteristics. Some caregivers (*n* = 3, 23.3%) indicated that people with MCI might turn down invitations to social events or other opportunities.

*‘People with MCI may evaluate the activities to see if they suit them. They may turn down the activities which [they consider to be] childish, [or which does] not match to their style.’ [Therapist 6].*

Some therapists suggested that educating people with MCI about the importance of attending social activities, which can enhance their external motivation and community acceptance, would be beneficial.

*‘We do a lot of social advocacies, including raising public awareness. It is to do advocacy in the community, and then [develop a] friendly cognitive disorder community, and then go to cooperate with different public welfare organizations.’ [Therapist 7].*

One therapist suggested that if an increasing number of citizens realized the importance of social participation and its role in preventing cognitive decline, the social environment would become increasingly friendly toward people exhibiting MCI symptoms. Therefore, a positive societal initiative would be to conduct science popularization programs related to cognitive disorders and/or the value of social participation to create an equal and friendly community atmosphere.

*‘I think the most important thing for the elderly in the community is to have more popularized content [regarding their condition], to let them [society] know which aspects are helpful to slow down their cognitive decline, which is very important.’ [Therapist 9].*

The therapists also mentioned that some people with MCI may be attracted to opportunities related to trade and production, such as handicrafts that could be sold for a profit. The economic value of the output could better attract them to participate in activities and provide a sense of fulfillment.

*‘Then let us make a bunch of flowers, and the flowers can be sold.’ [Therapist 1].*

#### 3.1.2. Mobility challenges and required support

Mobility issues continuously have a negative impact on life. Some caregivers (*n* = 5, 38.5%) and therapists (*n* = 5, 50%) reported that people with MCI might consider the distance needed for travel, whether the stairs at the location will be challenging, and the availability of the caregiver when determining whether to engage in activities.

*‘Obstacle for them may be the physical condition, [which is] is one aspect, that is, if the distance for him is a hindrance… [or] for example if he can walk to [the place], this ease of access to him is a bonus… if you need to take a ride in the car ah… or if this activity site [means he needs to go] up and down the stairs ah, for them all these things will be a hindrance.’ [Therapist 4].*

One therapist commented that people with MCI require more user-friendly facilities. Such facilities include friendly indoor moving lines designed to help people with MCI find the right direction or path.

*‘Physiological level, many times you may say I am not in the nutritional diet to do some changes, or I am in the spatial environment to give it a good room design barriers to do some good, or hardware may still be to take care of such elderly cognitive decline brought about by some obstacles, maybe slightly in color on the line of motion are to do changes.’ [Therapist 5].*

#### 3.1.3. Intensive grandparenting responsibilities

One respondent with MCI was responsible for caring for a spouse or grandchildren. As one therapist mentioned, people with MCI might isolate themselves from the outside world due to limited time, perhaps due to familial burdens or commitments. Individuals in these situations are likely to bury themselves in activities related to supporting dependent spouses or grandchildren and only have a few spare hours per day. They may live at the home of a child or live nearby. Before their grandchild is older enough (more than 3 years old) to go to kindergarten, they may spend 7 am-7 pm or longer with their grandchild since their son/daughter is working. When their grandchild goes to kindergarten, the caring time becomes shorter, it may last from 7 am-8 am, 3 pm-7 pm.

*‘I am in good health and have been helping my children with their children. After my grandson went to kindergarten, I only had some free time. Otherwise, I would have to watch him all the time. His parents were busy making money, and there was no time to watch.’ [FG7-P1].*

If their grandchild goes to primary school, they have one more responsibility on coaching assignments until their parents return home. Therefore, they may quit most of their social activities. As one person with MCI explained,

*‘My son has a 10-year-old 3rd grader [son] at home, he does homework every day, so if you spend more time on him, you will not have time to do these activities.’ [FG3-P4].*

The therapists suggested that family burdens could be reduced if people with MCI enjoy continual contact with family, friends, or others with MCI/dementia. These communication contexts, such as chatting during social activity breaks, help people with MCI better understand the challenges of others.

*‘I would suggest that the old man and other families who have the same experience should talk about their experiences from time to time, which would also relieve his pressure.’ [Therapist 7].*

Policy support was also mentioned. A therapist suggested that introducing relevant policy support to solve a family’s financial worries and ability to care for relatives with MCI would be beneficial. In Beijing, multiple institutions in the community offer many care services. However, not every family can afford these services. Consequently, informal caregivers are the most common source of care for people with MCI, placing a burden on family members. Policies targeted at supporting these family members would help them a lot:

*‘If our policy is very supportive, or if our society does a good job in caring for cognitive disorders, I think this support will also be a good boost to the service users. I think it will be a good promotion for the service recipients’ [Therapist 1].*

#### 3.1.4. Feeling excluded In a closed-minded community environment

Community acceptance is a key factor affecting people’s social connections with MCI. The caregivers and therapists reported that closed-minded community environments neglect the needs of people with MCI; some older adults may even isolate people with MCI. People with MCI may feel unsafe and excluded in this environment. Some of the therapists (*n* = 3, 30%) recognized this exclusion of people with MCI. One therapist said:

*‘If the older adults now also decline in vision, hearing, [or if] both decline in the case, you have to use a form of Internet, or a form of music, or a kind of vision decline with these the elderly… how can you [then] get him to participate in social activities?’ [Therapist 9].*

Some older adults may inadvertently isolate people with MCI due to the stigmatization of cognitive impairment. As reported by one caregiver, other older adults may consider people with MCI to be less cognizant.

*‘Some old people sometimes [appear to be] less cognizant; he also wants to come to participate, but the sober mind of the elderly, he dislikes too much, there may be some of those aspects [underpinning] his reason [not to participate].’ [FG1-C3].*

Thus, it is necessary to develop inclusive social environments in which people with MCI can feel safe and included. One therapist suggested that it is essential to encourage people with MCI to take the first step to join social activities, thereby overcoming the negative concept that ‘outside is too dangerous’. People with MCI may enjoy the interactions experienced in such an environment.

*‘Influencing each other, giving each other a more relaxed environment, a more trusting and loving environment, the whole may be adjusted.’ [Therapist 6].*

Another therapist suggested that a friendly social environment could help generate internal motivation through the participation of multiple individuals, encouraging people with MCI to continuously participate in social activities.

*‘As long as he can go out and integrate into … such a social group, in there to get a good acceptance, tolerance, and then [this will be] well received. The second time he should be better, and then coupled with the fact that everyone will call out to him from WeChat, or give a call, he will be more likely to go a second time. So, the first footsteps to act, or internal motivation, is important.’ [Therapist 8].*

### 3.2. Social participation

The top five social activities reported by people with MCI reported were participating in the singing group (*n* = 24, 77%), playing chess or mahjong (*n* = 19, 61%), joining the dancing group (*n* = 18, 58%), chatting with friends (*n* = 10, 32%), and traveling (*n* = 9, 29%). The singing group was the most popular activity since singing helps make people with MCI feel fresher and younger. The dancing group and party group were the most preferred community-based activities. People with MCI who live in the community attend the community dancing group, which is led by a group manager and takes place at a specific dance center near the community. People with MCI who live in a care center attend dancing activities in a care center regularly. Some retired older adults in the party group asked people with MCI to join them in party activities, watch lectures, or conduct group discussions. However, if people with MCI live in the care center or are of advanced age, the party group demonstrates an increased unwillingness to invite them to join. Most respondents with MCI (*n* = 23, 74.2%) are satisfied with their performance because they understand and accept that old age affects performance. None of the people with MCI reported any plans to join any new activity, highlighting the importance of others introducing new activities to them. Physical situations impact individuals with MCI the most as they desire to return to their younger selves with no pain. Some people with MCI stop doing the things they love, such as traveling or playing basketball, due to their declining physical condition.

#### 3.2.1. Lack of motivation to overcome the challenges of social participation

Self-efficacy determines the confidence levels of people with MCI in completing a social participation task or achieving personal goals related to social activities. Most therapists (*n* = 8; 80%) expressed that these unresolved issues may decrease patient self-efficacy (such as forgetting doctor appointments). When people with MCI experience these challenges in social activities, they may blame themselves, as reported by a therapist:

*‘I think he does not feel that something is wrong with himself, like he cannot do something anymore, or sometimes he has a greater forgetfulness, or sometimes there are things that he should be able to do, but he does not do them well enough, but I think it will have a greater impact on his self-confidence.’ [Therapist 5].*

Therefore, some people with MCI express little motivation for social participation or related achievements, which may reduce their attendance at social activities.

As one participant said,

*‘There is no special need. I can stay indoors for a month.’ [FG3-P4].*

One caregiver reported that people with MCI enjoy social activities relevant to their own era, that is, the era of their youth. Such generational content encourages expression, memory, and interaction among people with MCI. Thus, activities such as ‘red songs’ (a group singing traditional patriotic songs) are often effective in attracting people with MCI.

*‘They think that one of them is in a happy mood, and the other is because we sing songs of their era, so they… can recall things from the past.’ [FG7-C1].*

The ability to use props or tools can also influence motivation. These include instruments that are easy to handle with a few buttons. Operating such implements will reduce the negative feelings associated with the inability to operate complex tools. Moreover, when the tools are easy enough to use that every person can easily create a melody or piece of artwork, self-efficacy is enhanced. As one therapist explained,

*‘For example, there is a musical instrument, I will briefly say, they cannot play the instrument, but they can knock… each person in the hands of the instrument is also different, [just as] each person’s voice is different.’ [Therapist 7].*

The content and character of social activities also influence the motivation of people with MCI. The therapists mentioned that commonality helps individuals make new friends, especially if they have the same interests, background, or hometown.

*‘Both individualized and at the same time some commonalities, because it is most important to integrate into the group. If he feels safe, he will feel very comfortable, and he will not have that state of anxiety.’ [Therapist 6].*

Only moderately difficult activities improved motivation. People with MCI clearly expressed that they did not want to participate in childlike activities.

*‘I hope that various activities are more suitable for the characteristics of the elderly after the research of experts. Sometimes the handicraft activities are very much like those of kindergartens, and I am not too happy about it. I want activities that are more suitable for our mental and physical characteristics.’ [FG1-P1].*

#### 3.2.2. Lower social efficacy and higher performance expectation

Some people with MCI exhibit lower efficacy regarding their social competencies but retain high expectations. One of the participants mentioned that she thought each participant in the dancing group should dance gracefully; thus, she was anxious about her ability to perform.

*‘I wanted to join the dance team before, but I was afraid my zero-base performance would drag the team down.’ [FG1-P2].*

Some people with MCI (*n* = 4, 12.9%) considered many factors unsafe and may not overcome such factors. Accordingly, they tend to reduce interactions or refuse opportunities to visit outside or unfamiliar environments. For instance, some people with MCI are afraid of falling:

*‘I used to like to go out. Now that I’m older, I cannot walk anymore and I’m afraid of falling.’ [FG5-P3].*

Some therapists (*n* = 4, 40%) reported that people with MCI felt anxious in unfamiliar places besides the perceived risks of going outside. One therapist reported:

*‘This effect is very obvious because we also did have a few cases, from the beginning he may be particularly anxious, especially in a new environment, he will be particularly insecure, he will often think about going home, think about leaving.’ [Therapist 6].*

People with MCI may gain encouragement from good team leaders, as one person with MCI mentioned:

*‘[I will] exercise, if there is a leader who could lead and drive us to do it. We will exercise more positively.’ [FG4-P4].*

Therapists agree on the importance of involving leaders in social groups.

*‘There should be many key figures to drive the group, or to lead [the group], to lead these people gathered. Also play a role like a leader. To enable him to better integrate.’ [Therapist 8].*

#### 3.2.3. Impaired storytelling ability

Impaired storytelling abilities affect the retelling abilities of people with MCI; thus, such individuals have fewer opportunities to convey their thoughts or memories. One person with MCI mentioned that they face challenges when trying to retell their experiences.

Several respondents with MCI (*n* = 3, 9.7%) mentioned that they have a shorter attention span.

*‘It is as if the attention… will not know where to run, will not be able to remember, ah on the time of doing something, suddenly [you] do not know to think of something, and forget what they are doing now.’ [FG3-P1].*

Moreover, one participant reported struggling to retell stories related to their experiences. They want to explain what happened but cannot introduce the story clearly.

*‘Sometimes, I cannot retell clearly to my husband what I experienced today. The more worried I am, the more I cannot explain what just happened.’ [FG5-P2].*

Regarding the increasing desire of people with MCI to express themselves, therapists suggest that being accompanied by familiar people, engaging in familiar activity themes, and using familiar objects may encourage sharing stories.

*‘We will try to design some activities in his context and give him enough attention. We can see that the effect of contrast before and after is very good. Both individualized, and at the same time, some commonality, because it is most important to integrate into the group. If he feels safe, he will feel very comfortable, and he will not have that state of anxiety.’ [Therapist 6].*

#### 3.2.4. Insufficient ability to perform independently in singing and dancing

Some people with MCI (*n* = 5, 16.1%) reported that they could not perform independently in shows, such as dancing or singing solo; they needed some reference or support during the activities. For instance, some people with MCI reported difficulty keeping up with the action, especially during new things, such as learning a new movement or finding a particular page in the lyric book.

When people with MCI face challenges remembering lyrics in a group activity, they may follow others’ lead. For instance:

*‘If I sing solo, after all, I must memorize it, if you say follow everyone on it, well is a trick, do not grab the first line, he said he could remind him of the original [which can be] linked up.’ [FG3-P5].*

Some caregivers (*n* = 3, 23.3%) reported that people with MCI are liable to forget why they are in a particular place, which stops them from engaging in activities. A caregiver mentioned:

*‘Or some old people like to play chess, and then when he is playing chess, he will be attracted by other things and forget to play chess. For example, he will think ‘what am I doing now? How can I sit here? I do not play chess.’ He would immediately leave, and this has happened.’ [FG1-C1].*

The therapists also mentioned teaching people with MCI simple methods for improving their memory difficulties.

*‘It depends on the specific MCI patient, where he is troubled, if it’s the kind of memory decline where his short-term memory …is very troubled, then we go to help him to do the relevant [memory exercises] together with the attention to tell him some memory methods, to teach him to use some compensatory measures to learn to use that kind of simplified memory, and then some memory methods.’ [Therapist 7].*

### 3.3. Technology to support social connections and participation

Nearly half of our study participants do not use smartphones, and most live in a care center. Some participants (*n* = 15, 48.4%) reported not knowing how to use smartphones, whereas others (*n* = 15, 48.4%) reported needing help when using their smartphones. Only one participant reported that she could use smartphones without support. Some people with MCI (*n* = 2, 6.4%) said they are good at using the features they use frequently but may rapidly forget how to use some lesser-used features. Overall, people with MCI who live in the community have more technology needs (e.g., for booking a taxi) and digital literacy than those living in a care center. WeChat (*n* = 14, 45%) is used the most since, people with MCI contact their friends and family via their individual WeChat accounts, receive new information, and chat in WeChat groups. Moreover, the voice message feature of WeChat truly helps people with MCI with low literacy levels (i.e., they cannot use keyboards to type Chinese words since they have not learned Pinyin). Some participants (*n* = 3, 9.6%) used *Pinduoduo*^2^ (an online shopping software) to buy things. Use of this app inadvertently creates a social circle through which group purchases can be conducted, as group buys have discounted prices. *Pinduoduo* has a feature through which individuals invite their friends to bargain, facilitating communication. Therefore, people with MCI contact friends more and invite them to use this software due to its mutual benefits. Additionally, taxi-hailing applications support participants in participating in social activities. Learning how to effectively use smart devices is an important first step. Some people with MCI (*n* = 4, 12.9%) reported learning how to use smartphones and applications through volunteers or their children.

#### 3.3.1. Lack of motivation for independent use of mobile phones

Motivation for independent mobile phone use is a key attribute affecting the learning behavior of people with MCI and depends on perceived benefits, costs, the original plan, and social influence. All participants who knew how to use smartphones reported perceived benefits, such as the WeChat call being free and stable and the car-hailing services being convenient.

*‘Compared with phone calling, the WeChat calling it is free and stable, and I can use the camera to see my friend online.' [FG4-P1]*

However, people with MCI who know how to use smartphones mentioned usability issues, such as pressing the wrong button, going to the wrong page, or encountering mobile phone viruses.

*‘Anyway, now it is WeChat, such as who I send a WeChat, answer a phone call. If I knew anything else, I would not. Just tell me when you download it, but you’ll forget it later, and you will not know how to click it when you pick up the phone. I do not know how to use shopping, but I’ll forget it soon after I’ve been taught.’ [FG3-P4].*

Misleading information is the main concern preventing the frequent use of technology. Participants expected that the mobile phone could identify and block misleading information, but this is not true.

*‘But now the things on this phone are also quite complicated, some people have ulterior motives to carry out unreasonable propaganda, I am often criticized by my daughter for believing this.’ [FG1-P3].*

In contrast, people with MCI who did not know how to use smartphones reported less context, opportunities, and independence. The therapists reported that people with MCI who live in sheltered communities might benefit from technology because they have more opportunities to use it. These individuals have more helpers, such as informal or formal caregivers, to accompany them to travel or visit other places.

*‘If people with MCI live in a [sheltered] community, they tend to be good at using WeChat. However, people with MCI who live in a care center have fewer use opportunities to use smart phones.’ [Therapist 5].*

Nearly half of the therapists (*n* = 4, 40%) and one caregiver (*n* = 1, 7.6%) noted that a lack of digital literacy might hinder people with MCI from going outside alone because they may need special support in some circumstances.

*‘In fact, they feel the difficulty is in, for example, the use of some intelligent devices, such as smartphones, computers… he wants to browse a web page, he will not, for example, some of them will, but he will press the wrong point wrong, and then there will be a lot of viruses on the phone, they just cannot get this kind of trouble more.’ [FG1-C2].*

In conclusion, some people with MCI do not recognize the benefits of using smartphones, and few use them; however, a lack of digital literacy may further hinder social interaction.

Therefore, the therapists suggested more customized digital literacy lectures or workshops reveal the benefits of technology and motivate people with MCI to learn how to use it.

*‘We should contact more communities to organize common software training for older adults to let them experience the real benefits of using technology. Then, they will be more motivated to learn and use it.’ [Therapist 5].*

#### 3.3.2. Negative social influence of using technology

Although technology is beneficial, some people with MCI (*n* = 4, 12.9%) fear the risk of fraud and unwanted privacy disclosures. One MCI patient mentioned her daughter criticizing her smartphone views:

*‘But now the things on this phone are also quite complicated; some people have ulterior motives to carry out unreasonable propaganda, I am often criticized by my daughter for believing this.’ [FG1-P3].*

Some caregivers (*n* = 2, 15.4%) and therapists (*n* = 5, 50%) emphasized that fictitious and misleading online information can threaten older adults because they have limited judgment. Moreover, one therapist mentioned that people with MCI complained that technology feedback could feel cold, and they preferred interacting with real people.

*‘We tried some little games, too, and they worked fine. But the feedback from the machine is cold; it’s just a number. And it simply says, “Oh, you did great. You beat the game.’ [Therapist 7].*

One of the therapists suggested that if people with MCI receive sufficient encouragement to learn technology and the benefits derived from technology from friends and family, they will keep learning.

*‘I think… if he is encouraged to go to one, and then let him get some pleasure from it, so that he can experience this sense of integration, coupled with the encouragement of family, relatives, and friends. Their support should really do to promote this behavior.’ [Therapist 8].*

## 4. Discussion

Despite the importance of social participation in daily living, little is known about the level of social participation in people with MCI. There is a specific lack of understanding regarding barriers to social connections, social participation, and the use of technology. Consequently, there is a lack of effective recommendations for overcoming the social participation barriers of people with MCI. Thus, this study research represents an urgent preliminary step toward a more profound understanding of social participation among people with MCI.

### 4.1. Barriers and recommendations for establishing social connections

In this study, we have identified several barriers that may impede the ability of individuals with MCI to establish social connections. These barriers include limited socialization opportunities and suitable activities, challenges related to mobility and required support, intensive grandparenting responsibilities, and feelings of exclusion within closed-minded community environments. Our findings indicate that people with MCI who live in a community and a care center have no difference in social activity type. The available activities for individuals with MCI primarily revolve around solo, literary, and artistic pursuits, which may not cater to the needs of those lacking expertise in these areas. As a result, some individuals with MCI may decline participation in such activities. To address this issue, we explore various possibilities based on scientific methodologies and theories to organize various social activities that encourage individuals with MCI to venture outside their homes. Additionally, mobility difficulties, which are crucial for maintaining independent living among older adults (72), can further hinder their participation. Consequently, individuals with MCI rely on support from others and the accessibility of facilities. Moreover, the presence of closed-minded community environments within neighborhoods also poses environmental barriers for individuals with MCI who live in a community. Consistent with previous research, individuals with MCI may face stigma (73). To overcome these challenges, it is essential to establish additional services that support older individuals with mobility issues, improve the accessibility of facilities, and foster a welcoming social environment. These measures may help generate internal motivation through increased participation, thus encouraging individuals with MCI to engage in social activities continuously.

Apart from the barriers within the community, families also pose a significant obstacle, namely the burden of family responsibilities, which emerged as an unexpected finding in our study. Some people with MCI in our study have an intensive family burden before they move to a care center. Previous research has highlighted the positive impact of family responsibilities, such as grandparenting, as it provides individuals with MCI with a new social role (64). However, our participants reported that due to their spouse’s dependency or their children’s inability to care for their grandchildren, many individuals with MCI, particularly female respondents, find themselves shouldering additional family responsibilities. Cultural reasons and financial constraints may prevent these families from affording expensive childcare services, thereby increasing the reliance on grandparents. While this responsibility offers a renewed sense of social connections, it also brings new pressures. Engaging with grandchildren can assist in re-establishing lost social connections (63), and paradoxically, spending more time caregiving for grandchildren has been shown to benefit the psychological health of Chinese-American grandparents, supporting the role enhancement theory (74). However, these efforts demand significant time and energy, leaving individuals with MCI with limited opportunities to explore their own interests. Moreover, the combination of advanced age and a high burden of caregiving for grandchildren accelerates the decline in global cognitive function and episodic memory among these grandparents (66), aligning with previous studies in China that highlight the accelerated cognitive decline resulting from high-intensity care for younger grandchildren (75). Therefore, individuals with MCI should maintain personal social activities to protect against further cognitive decline. While grandparenting can be a fulfilling social role, monitoring and managing suitable grandparenting loads is vital to ensure continued social participation.

### 4.2. Barriers and recommendations for social participation

During our study, we also identified barriers that affect the social participation process for individuals with MCI. These barriers include the inability to perform independently in social activities, lower social efficacy, higher performance expectations, limited motivation for social challenges, and impaired storytelling abilities. Previous research has shown that the impact of social resources on loneliness can mitigate cognitive impairment (76). Our findings help explain this phenomenon by highlighting how memory decline hinders individuals from recalling and fluently retelling recent events or stories, thus impairing their ability to perform independently. This effect can lead to stigma and avoidance of social participation opportunities, no matter people with MCI is live in a community or in a care center. We found that individuals with MCI tend to have higher expectations of social performance and fear that their behavior, which may not meet their own expectations, will negatively affect others. This finding aligns with previous studies linking self-efficacy to social participation (77) and sheds light on why individuals with MCI may decline social engagement. Setting appropriate goals can help moderate high or unrealistic expectations and mitigate these challenges. Moreover, individuals with MCI may experience lower social participation achievements, reduced social efficacy, and higher expectations due to difficulties in achieving desired outcomes (78). Building self-efficacy is crucial in addressing this issue, as it supports older adults in overcoming psychological barriers (79). Therefore, it is important to help individuals with MCI manage their expectations and enhance their social efficacy.

Furthermore, we found that storytelling challenges have a more pronounced impact on social participation. Tasks involving story retelling can distinguish individuals with MCI from cognitively healthy adults, as they may exhibit differences in nonword repetition, story grammar, and narrative comprehension scores (80, 81). This effect is particularly relevant to the subtype of MCI known as amnestic MCI, characterized by memory complaints (2), and language impairment can further hinder the social participation process. Individuals with MCI often struggle with comprehending complex language, which impairs their ability to engage in daily verbal interactions (82). The difficulties they experience in expressing themselves linguistically can undermine their confidence and lead to avoidance behaviors, exacerbating the deterioration of their language skills. Meaningful interventions should therefore incorporate activities aimed at improving language function. Previous research has explored the effectiveness of storytelling activities in improving language function through book/video/game workshops (83–85), tablet applications (86), and language and communication interventions (87). Storytelling is also beneficial in reminiscence therapy (88). However, existing interventions, such as workshops, often require a high level of digital literacy for participants to edit video or audio clips, which may not be accessible for individuals with MCI in China. Digital interventions also involve memory-based actions that may be unfamiliar and challenging for Chinese individuals with MCI. Therefore, it is necessary to further investigate accessible technologies with suitable stimuli that can help individuals with MCI share their stories effectively.

### 4.3. Barriers and recommendations for the use of technology for social connections and participation

We also examined the overall status of technology use for social connections and participation among individuals with MCI. People with MCI who live in a community have more chances to use technology for social connections and participation. Our findings align with previous research indicating that the prevalence of technology use among people with MCI is low (89). According to the 49th Statistical Report on China’s internet Development released by the China Internet Information Center in February 2022, individuals aged 60 and above accounted for 11.5% of total internet users, with a 1.2% increase since June 2020. The internet use rate for this age group reached 43.2% (90). The report also highlighted that 99.5% of older internet users access the internet through mobile phones, primarily using application software. In our study, we recruited a higher proportion of older mobile phone users compared to previous investigations. These mobile phone users tend to be younger, live in the community, and have a higher educational background. Consequently, participants with these characteristics may have more opportunities to use mobile phones, greater motivation to utilize them, and access to resources for learning how to use smartphones. However, limited digital literacy remains a challenge for smartphone use, potentially impeding older Chinese adults from embracing digital technologies (91). Several programs, such as age-on learning workshops (92), group courses on social networking sites (93), and technology-learning education programs, have been designed to enhance digital literacy among older adults (26, 94), particularly in everyday technologies like smartphones (95). However, these programs are often standalone and lack the continuous and on-demand support required by people with MCI. Moreover, they may not adequately address the motivation to use mobile phones, which represents a significant barrier to technology adoption.

In our study, we identified the motivation for independent mobile phone use and the negative social influence of technology use as additional barriers. We propose that the motivation for independent mobile phone use among people with MCI comprises perceived benefits, social impact, costs, and the influence of the original scheme. The benefits include psychological advantages such as independence and self-esteem, as well as improvements in cognitive abilities and skills. Costs encompass purchase costs, learning costs, and concerns related to privacy. The effect of the original scheme refers to the previous plan or approach used, which may be influenced by environmental changes, such as the Health Code requirement during the COVID-19 pandemic. Social impact represents the influence of individuals surrounding people with MCI, including both supportive and opposing attitudes toward technology use. If individuals have a positive motivation to learn and use technology, they are more likely to acquire the necessary skills. However, if their motivation is negative, they are likely to resist learning and using technology. Unlike previous studies, our findings shed light on children’s concerns regarding technology usage. Social influence from family members was found to impact technology usage behavior, both positively and negatively. While some family members support technology use, others perceive it as increasing the risk of online fraud for people with MCI. Nonetheless, social pressures from family and friends do not significantly predict technology usage (96). Therefore, we recommend enhancing the perceived benefits of smartphone use, reducing learning costs, and collaborating with partner institutions to promote technology adoption.

### 4.4. Interconnections between barriers and recommendations to social connections, social participation and the use of technology

**Figure 1:**
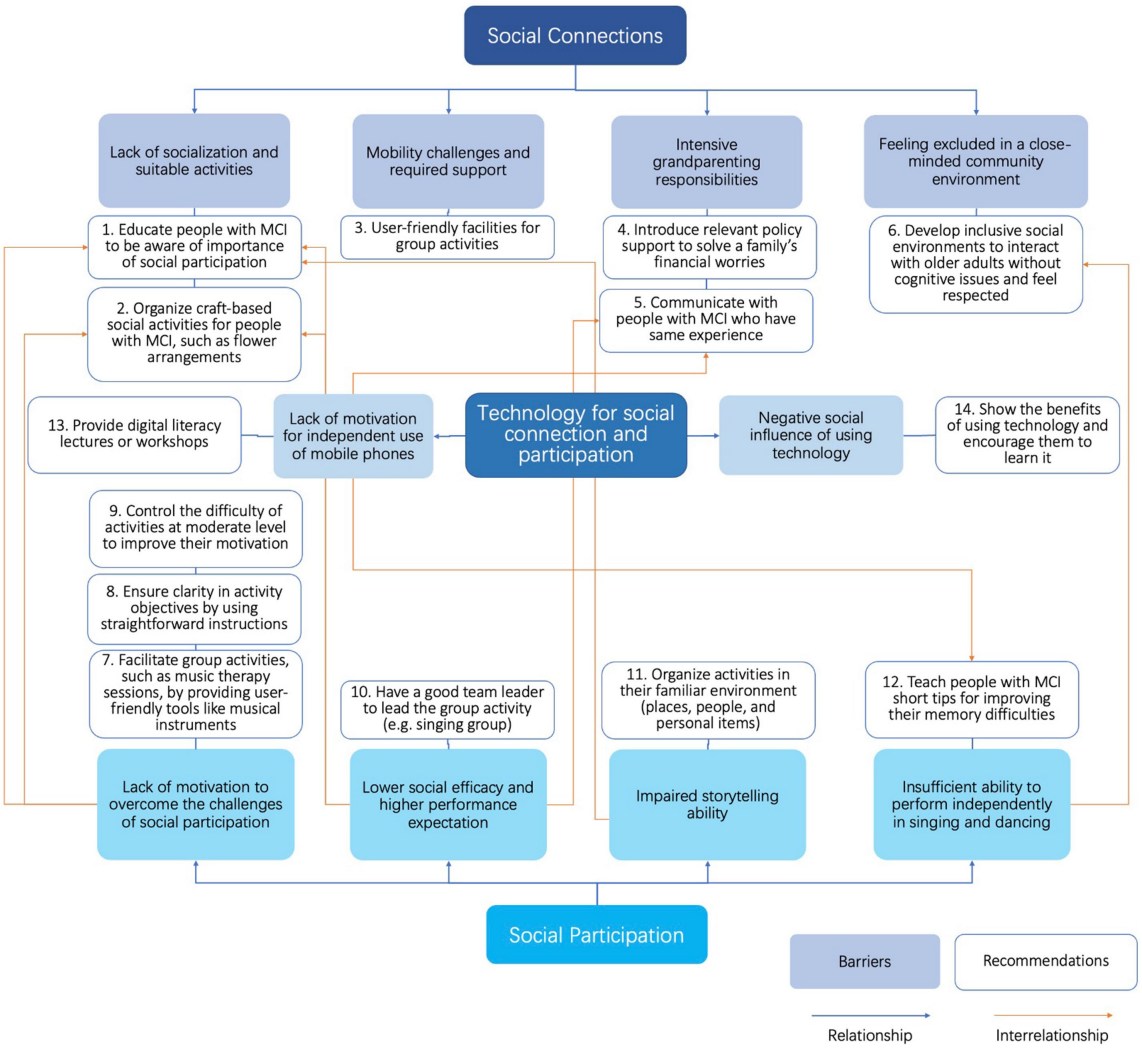
Interconnections between barriers and recommendations to social connections, social participation, and use of technology.

As depicted in Figure 1, our research findings provide a comprehensive summary of recommendations to assist individuals with MCI and practitioners in overcoming these barriers. The lack of motivation to overcome social participation challenges and lower social efficacy exhibit similar recommendations to address the barriers to socialization and suitable activities in social connections. Introducing attractive and engaging activities can catalyze to motivate individuals with MCI. Additionally, overcoming barriers to social connections and participation can positively impact on the adoption of mobile phone technology. Communication with other individuals with MCI and memory-enhancement techniques can facilitate learning mobile phone skills. Thus, an interrelationship exists among social connections, social participation, and technology adoption regarding barriers and recommendations. To enhance social connections and participation among people with MCI, it is crucial to consider these three concepts holistically within technology-based interventions. By integrating social connections, participation, and technology support, we can design more effective interventions to improve the overall well-being and quality of life for individuals with MCI. Consistent with previous studies (20, 97, 98), our study confirms that a significant number of people with MCI experience reduced social connections and participation. Additionally, we observed a disparity in satisfaction levels between people with MCI and their caregivers regarding participation. These findings support the conclusions of Donkers who reported that individuals with MCI expressed satisfaction with their current social participation levels (99), while therapists and caregivers expressed dissatisfaction. The tendency of people with MCI to underestimate their abilities and set low goals contributes to their overall contentment with their current level of social participation. These collective findings highlight the challenges faced by individuals with MCI in establishing social connections, engaging in social participation, and effectively utilizing technology.

### 4.5. Framework to enhance social connections and social participation

**Figure 2:**
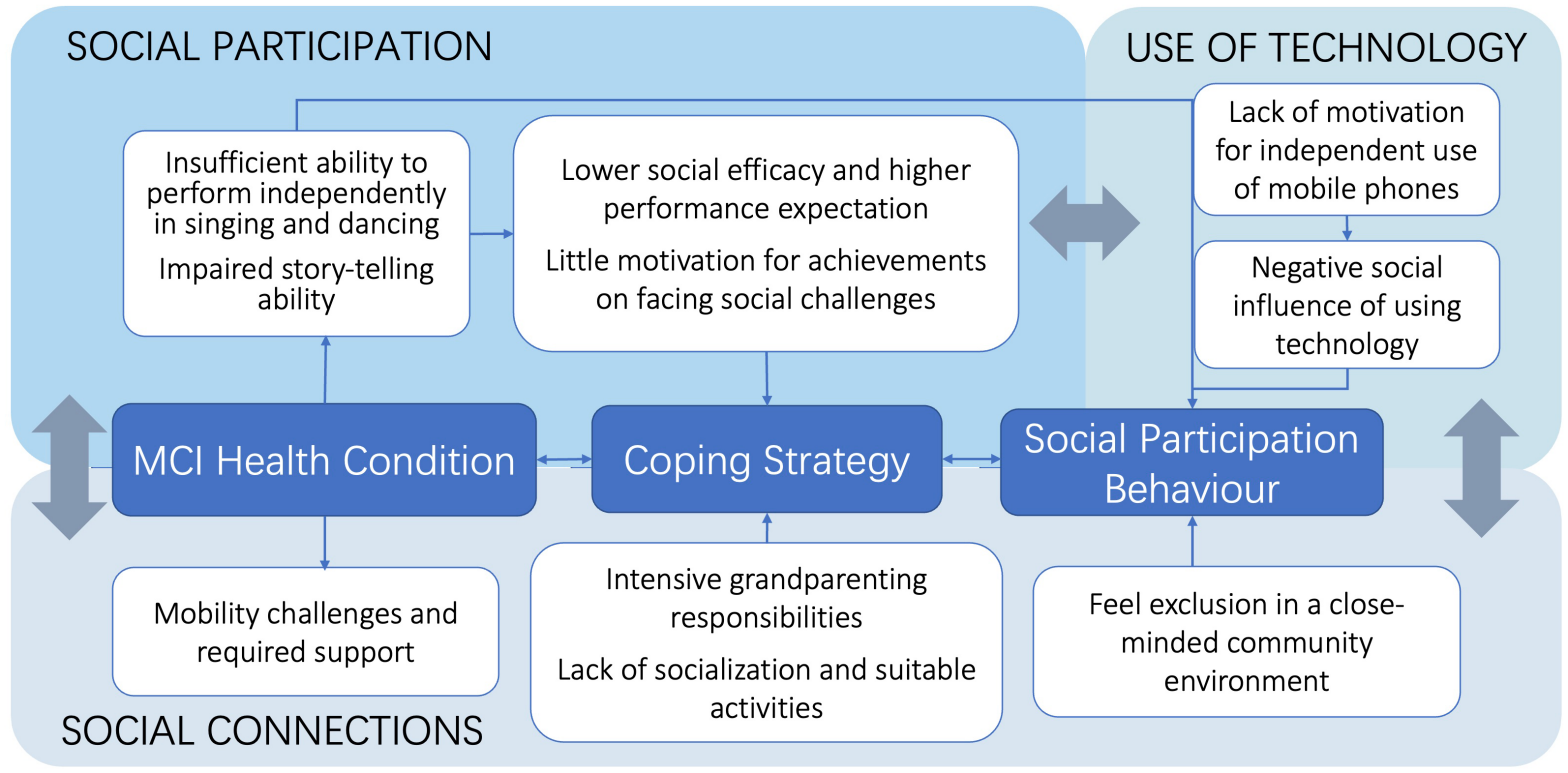
Framework to enhance social connections and social participation and the role of technology among people with MCI.

The main theoretical structure employed herein has been adapted from the International Classification of Functioning, Disability and Health (ICF) model, which provides six categories of factors (100), supplemented by the model of the effect of social participation on health (101). Building on these, and in combination with our findings, we propose a novel theoretical framework of social connections, social participation, and technology usage for people with MCI (see Figure 2).

We maintain the core structure of health conditions, combined activities, and behavior within the social participation behavior element of the ICF model (100). Moreover, we combined informal social participation and volunteering in a model of the effect of social participation on health (101). Amano identified three patterns of social participation (informal only, formal and informal, and low social involvement) among people with MCI (97); our research confirms that most people with MCI experience the same barriers. Some people with MCI may use more positive coping strategies, whereas others may use more negative ones. Thus, people with MCI maintain diverse and variable social participation patterns. Accordingly, it is necessary to extrapolate the nuances of these barriers while distinguishing among social participation patterns. Lydon clustered factors into contextual groups (environmental factors, personal factors) and health factors (cognitive, emotional, and physical factors) (31). These factors affect social connections and participation. Our framework confirms the relationship between these factors and social connections and participation. Townsend reviewed barriers and facilitators for older adults and reported that information and communication technology (ICT) factors are individual factors (102). However, we found that technology usage barriers are associated with social connections and participation barriers since technology supports all types of interaction (103).

If people with MCI learn to master social media, they can more readily contact family and friends, significantly reducing social distance. Likewise, if people with MCI master navigation or taxi-hailing apps, they can visit more places independently. In addition, social connection factors impact social participation factors. Given that people with MCI have fewer body function issues, we combined body functions with health conditions into the overarching MCI health condition. Moreover, we introduce the coping strategy between the health condition and social participation, such as acceptance, positive reframing, and staying connected through technology (104). Behavioral or cognitive coping strategies are attitudes developed through life experience and aim to reduce the functional effects of an impairment or disability, enabling people with MCI to continue participating in their social environment (105). Mobility challenges inevitably influence these coping strategies since higher instances of mobility impairment among Chinese elderly adults make them more likely to report lower life satisfaction, partly because they often engage in fewer social activities (106).

Our findings suggest that interventions to create new types of social activities that increase social participation will likely be useful for people with MCI. We found that personal factors affect social participation more than environmental factors. Therefore, it is important to help people with MCI rebuild their self-efficacy and confidence, and managing expectations through psychological services or lectures is important. The government and relevant policymakers should consider multidimensional initiatives to, for instance, protect people with MCI from heavy family burdens, introduce policies to guarantee a certain duration of social activity and support families with dependent older adults with MCI.

### 4.6. Strengths and limitations

This study’s strength is the collection of three perspectives, the people with MCI and their caregivers and therapists. We summarized the barriers and recommendations for social participation based on multiple angles. Online focus groups might reduce the data quality because we relied on the caregivers to interpret some participants’ opinions as we could not hear them clearly. We conducted convenience sampling; our participants were people with MCI who voluntarily participated in this study and were optimally connected with services through institutions. These participants had an advantage over those who could not voluntarily participate. Thus, these individuals may experience fewer barriers or have more positive coping strategies. Furthermore, these barriers and suggestions would be useful to older adults since they may meet the same challenges in social participation but more slightly, and less cognitive issues. As the review paper identified individual factors [health, motivation, and Information and Communication Technology (ICT)], and environmental factors (transport and accessibility and individual living situations) (102). People with MCI and older adults have common challenges on motivation, ICT, and transportation.

## 5. Conclusion

This study aimed to identify and ascertain the barriers to and recommendations for improving people’s social connections and participation with MCI. The results generated herein reveal that people with MCI may experience obstacles originating from themselves, their families, and society. The insights gained from this study will assist in designing future interventions to enhance social participation, such as developing interventions to support storytelling activities. We should explore new activities that attract more people with MCI, enhance their cognitive abilities, and improve the accessibility of technology-based interventions. We should protect people with MCI from taking on too many family responsibilities and social stigma. Therefore, the government should consider introducing relevant support policies and building age-friendly environments.

## Data availability statement

The raw data supporting the conclusions of this article will be made available by the authors, without undue reservation.

## Ethics statement

The studies involving human participants were reviewed and approved by Swinburne University Human Research Ethics Committee (SUHREC). The patients/participants provided their written informed consent to participate in this study.

## Author contributions

AA contributed to the conception and design of the study. DZ collected the data, conducted the data analysis with the guidance of AA, and wrote the first draft of the manuscript. AA and WL supervised the project and assisted in rewriting several sections of the manuscript. All authors contributed to the manuscript revision, read, and approved the submitted version.

## Conflict of interest

The authors declare that the research was conducted in the absence of any commercial or financial relationships that could be construed as a potential conflict of interest.

## Publisher’s note

All claims expressed in this article are solely those of the authors and do not necessarily represent those of their affiliated organizations, or those of the publisher, the editors and the reviewers. Any product that may be evaluated in this article, or claim that may be made by its manufacturer, is not guaranteed or endorsed by the publisher.

## Footnotes

^1^ https://www.deepl.com/translator

^2^ https://www.pinduoduo.com/
