# Supplementary material for: Social connections and participation among people with mild cognitive impairment: barriers and recommendations
Source: Front Psychiatry. 2023 Jul 5;14:1188887. doi: 10.3389/fpsyt.2023.1188887 (PMC10356108; doi:10.3389/fpsyt.2023.1188887)
Supplement: Supplementary file 2 [file Table_2.docx]

### Appendix 2 1-on-1 interview guidelines (therapists).

| Topic | Number | Question for therapists |
| --- | --- | --- |
| Basic Information | 1 | How long are you being a therapist? |
|  | 2 | What intervention you usually provide? |
| Instrumental activities | 3 | What instrumental activities of daily life are difficult for people with MCI? |
| Social Connection | 4 | What social connection circle you suggest them to maintain? |
|  | 5 | If you want to enhance the social connection of people with MCI, what would you suggest them to do? |
|  | 6 | In your opinion, what may stop them to build social connection |
|  | 7 | In your opinion, what may motivate them to build social connection |
| Social Participation | 8 | Which social activities are most popular? |
|  | 9 | What attributes of social activities will attract them? |
|  | 10 | What kind of social activities you suggest them to do more of? |
|  | 11 | What difficulties do they have in doing these activities? (e.g., memory, language, attention. Visuospatial, executive/planning) |
|  | 12 | What will you suggest them to cope with these difficulties? |
|  | 13 | What instrumental activities of daily life are difficult for people with MCI? |
|  | 14 | What will you suggest them to change first? |
| Community-based activities | 15 | Which social groups are most popular? |
|  | 16 | Where are the social groups happening? |
|  | 17 | Which kind of social group you suggest them to join? |
|  | 18 | How the participation generates the sense of belonging? |
| Closing Question | 19 | Do you have anything to further explain? |
