# Supplementary material for: Social connections and participation among people with mild cognitive impairment: barriers and recommendations
Source: Front Psychiatry. 2023 Jul 5;14:1188887. doi: 10.3389/fpsyt.2023.1188887 (PMC10356108; doi:10.3389/fpsyt.2023.1188887)
Supplement: Supplementary file 3 [file Data_Sheet_1.docx]

# Appendix

### Focus group guidelines (people with MCI and caregivers).

| **Topic** | **Number** | **Question for people with MCI** | **Question for caregiver** |
| --- | --- | --- | --- |
| Basic Information | 1 | How long was your diagnosis with MCI? | What is the relationship between you and the patient? |
|  | 2 | NA | How long are you a formal/informal caregiver? |
| Daily routine | 3 | Could you please tell me about your typical day? What do you usually do? (The daily routine exercises) | NA |
|  | 4 | What do you do around your home (e.g., go to a food market, pick up your grandchild, clean, cook, walk, square dance, manage finances)? | What do older adults tend to do around their home (e.g., go to a food market, pick up a grandchild, clean, cook, walk, square dance, manage finances)? |
| Social Connection | 5 | Could you please tell me your social circle? | Which level of social connection circle do you suggest them to expand? |
|  | 6 | How do you usually connect with your family, friends? | If you want to enhance the social connection of people with MCI, what would you suggest them to do? |
|  | 7 | What kind of technology support you in managing your social connection? | What kind of technology support older adults in managing social connection? |
|  | 8 | How much effort do you put on? | How much effort do they put on? |
|  | 9 | What may stop you from building social connections? | In your opinion, what may stop them from building social connections? |
|  | 10 | What may motivate you to build social connections? | In your opinion, what may motivate them to build social connection |
| Social Participation | 11 | What social activity do you usually do? | Which social activities are most popular? |
|  |  | Are you satisfied with the things you do? Why? | What attributes of social activities will attract them? |
|  | 12 | Are there some social activities you would like to start doing, resume doing, or do more of? | What kind of social activities do you suggest them to do more often? |
|  | 13 | What difficulties do you have in doing these activities? (e.g., memory, language, attention. Visuospatial, executive/planning) | What difficulties do they have in doing these activities? (e.g., memory, language, attention. Visuospatial, executive/planning) |
|  | 14 | How will you cope with your difficulty? | What will you suggest to them to cope with these difficulties? |
|  | 15 | What kind of technology support you in managing your social participation? | What kind of technology support older adults in managing their social participation? |
|  |  | How does it help？ | How does it help？ |
|  | 16 | If you woke up tomorrow and did not have any difficulties, what would be different? | NA |
|  | 17 | As you mentioned, what do you want to change most? | What will you suggest them to change first? |
| Community-based activities | 18 | Do you join social groups or clubs? | Which social groups are most popular? |
|  | 19 | Where are the social groups happening? | Where are the social groups happening? |
|  | 20 | How do you evaluate the social group that is suitable for you? | Which kind of social group do you suggest them to join? |
|  | 21 | How do you feel when you are in these social groups? Could you describe it? | How the participation generates a sense of belonging? |
| Closing question | 22 | Do you have anything to explain further? | Do you have anything to explain further? |

### One-on-one interview guidelines (therapists).

| **Topic** | **Number** | **Question for therapists** |
| --- | --- | --- |
| Basic Information | 1 | How long are you being a therapist? |
|  | 2 | What intervention do you usually provide? |
| Instrumental activities | 3 | What instrumental activities of daily life are difficult for people with MCI? |
| Social Connection | 4 | What social connection circle do you suggest them to maintain? |
|  | 5 | If you want to enhance the social connection of people with MCI, what would you suggest them to do? |
|  | 6 | In your opinion, what may stop them from building social connection. |
|  | 7 | In your opinion, what may motivate them to build social connection |
| Social Participation | 8 | Which social activities are most popular? |
|  | 9 | What attributes of social activities will attract them? |
|  | 10 | What kind of social activities do you suggest them to do more often? |
|  | 11 | What difficulties do they have in doing these activities? (e.g., memory, language, attention. Visuospatial, executive/planning) |
|  | 12 | What will you suggest to them to cope with these difficulties? |
|  | 13 | What instrumental activities of daily life are difficult for people with MCI? |
|  | 14 | What will you suggest them to change first? |
| Community-based activities | 15 | Which social groups are most popular? |
|  | 16 | Where are the social groups happening? |
|  | 17 | what kind of social group do you suggest them to join? |
|  | 18 | How the participation generates a sense of belonging? |
| Closing Question | 19 | Do you have anything to explain further? |
